# Cerebrovascular Events After Transcatheter Aortic Valve Implantation

**DOI:** 10.3389/fcvm.2018.00104

**Published:** 2018-07-31

**Authors:** German Armijo, Luis Nombela-Franco, Gabriela Tirado-Conte

**Affiliations:** Interventional Cardiology Department, Cardiovascular Institute, Hospital Clinico San Carlos, Instituto de Investigación Sanitaria San Carlos (IdISSC), Madrid, Spain

**Keywords:** TAVI, cerebrovascular event, stroke, aortic valve stenosis, transcatheter aortic valve implantation, transcatheter aortic vave replacement

## Abstract

Transcatheter aortic valve implantation (TAVI) has emerged as an alternative less invasive treatment for patients with symptomatic severe aortic stenosis. Despite the technological development and knowledge improvement in recent years, neurological complications remain a concern, especially with the expansion of the technique toward younger and lower risk patients. Clinical cerebrovascular events have an important impact on patients' morbidity and mortality with a multifactorial origin. While cerebral microembolizations during TAVI is a universal phenomenon and embolic protection devices have been developed in an attempt to reduce them, their clinical utility remains unclear. We review the current evidence on cerebrovascular events associated with TAVI and potential preventive strategies.

## Introduction

Transcatheter aortic valve implantation (TAVI) has been established as the therapy of choice in patients with severe aortic stenosis of high or prohibitive risk, and in the last years as a valid alternative to surgery (SAVR) in patients with intermediate risk (1–4). Despite the great technological advances, cerebrovascular events (CVE) remain one of the most feared complications, increasing the risk of morbidity and mortality at short and long term (5, 6). The incidence of CVE following TAVI varies according to definition ranging from 1 to 11% (7) and with a similar frequency compared to SAVR in randomized clinical trials (4, 8). However, it exceeds any other daily percutaneous cardiac intervention especially in the acute period to decrease later in the following months (6, 9). Despite clinically strokes represents only a small proportion of patients, silent cerebral embolisms are an almost universal finding associated with this procedure. Furthermore, the real impact of these micro emboli on patients' cognitive function and development of future cerebral complications remain unclear. We present a review of the current knowledge about CVE following TAVI and insights about potential preventive strategies and future implications.

## Classification and definition of cerebrovascular events

In an effort to unify the discrepancies in the stroke definition used across the studies, the Valve Academic Research Consortium (VARC-I) in 2012 recommended to use the definitions of transient ischemic attack (TIA) and stroke (10). TIA was defined as a neurological deficit that resolves rapidly, in <24 h, without evidence of tissue injury in neuroimaging study. Stroke was defined as a new focal or global neurological deficit that persisted more than 24 h, or <24 h associated with cerebral injury in neuroimaging study, or if the neurological deficit resulted in death. The severity of stroke is usually categorized according to the modified Rankin Scale (mRS), classifying it into disabiling (major stroke mRS ≥2) and non-disabiling (minor stroke mRS <2) (Table 1). This criteria have been recently complemented by the Neurologic Academic Research Consortium in 2017 after the preparation of a consensus document where they established a new classification and also defined the endpoints applicable to clinical trials (12) (Table 2).

**Table 1:** The modified Rankin Scale for classification of stroke severity.

**Severity**	**Degree of neurological damage**
Level 0	No disability: no restriction of usual activities
Level 1	No significant disability: able to carry out all usual activities despite neurologic deficits
Level 2	Slight disability: able to look after own affairs without assistance but is unable to carry out all previous activities
Level 3	Moderate disability: requires some help but is able to walk without any assistance
Level 4	Moderately severe disability: cannot to attend to own bodily needs without assistance or requires assistance to walk
Level 5	Severe disability: requires constant nursing care and attention
Level 6	Death

*Adapted from Sacco et al. (11)*.

**Table 2:** Cerebrovascular events definitions according to the Neurological Academic Research Consortium (2017).

**NEUROARC NEUROLOGICAL EVENT DEFINITIONS**		
Type 1	Overt injury	Ischemic stroke
		Cerebral / Subarachnoid hemorrhage
		Hipoxic Injury
Type 2	Covert injury	CNS Infarction
		CNS Hemorrhage
Type 3	Symptoms without injury	TIA
		Delirium
**CLASSIFICATION OF NEUROLOGICAL EVENT TIMING**		
Periprocedural	<30 days post-intervention	
Late	>30 days post-intervention	

*CNS, central nervous system. Adapted from Lansky et al. (12)*.

## Incidence of clinical cerebrovascular events

**Figure 1:**
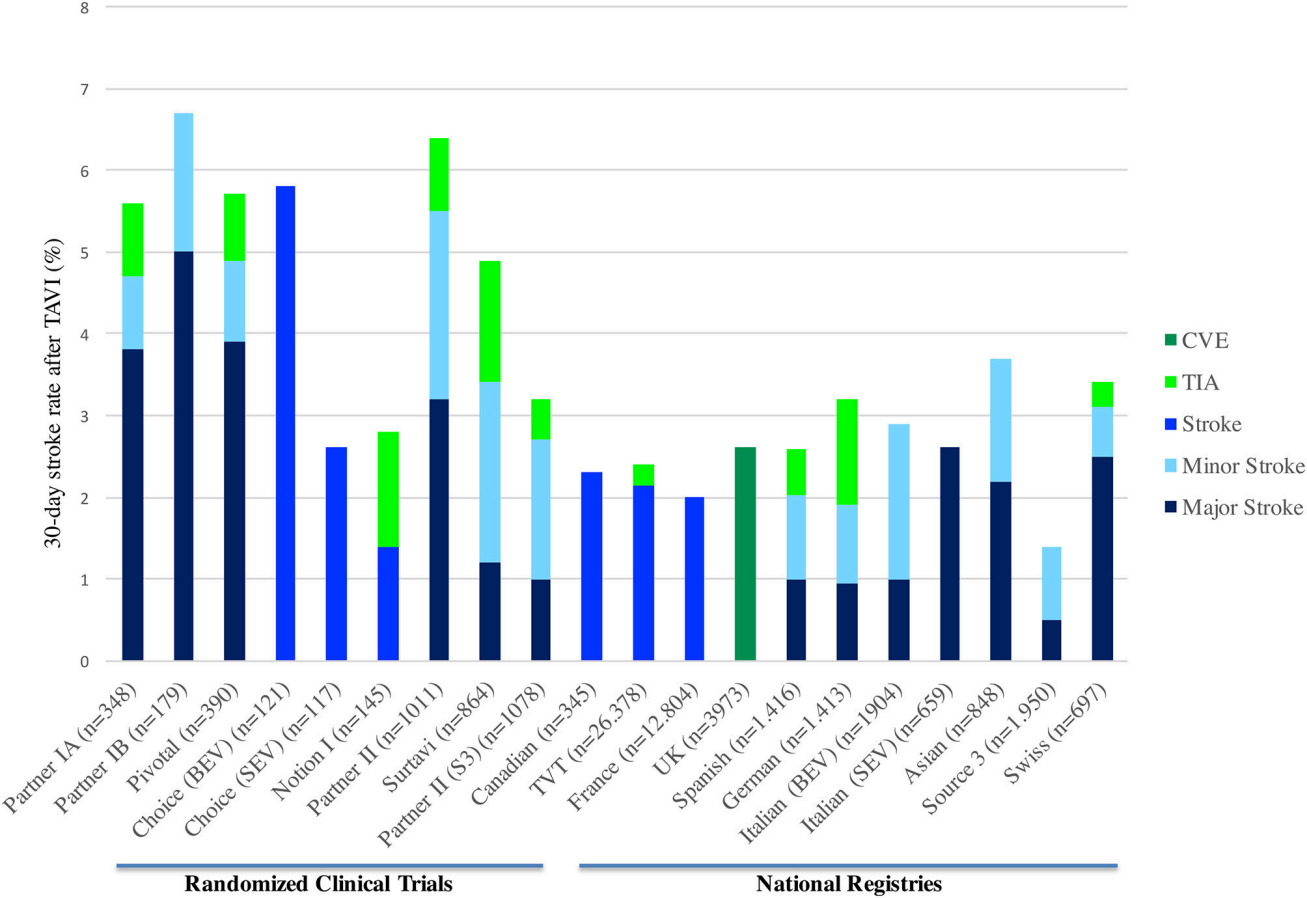
Thirty-day stroke rate in randomized clinical trials and TAVI national registries. CVE, Cerebrovascular events; TIA, Transient ischemic attack; TAVI, transcatheter aortic valve implantation; BEV, Balloon-expandable valve; SEV, Self-expandable valve.

Cerebrovascular complications related to TAVI showed a significant variability between centers and studies, ranging from 1 to 11% (7). Rates of 30-day CVE in randomized trials and national registries are shown in Figure 1. This variability might be explained depending on the definition used, study design, diagnostic methods, patient risk-profile, site-specific factors, and systematic evaluation by a neurologist (13–15). The stroke incidence reported in most studies was generally a combination of non-disabling (*minor*) and disabling (*major*) stroke, while TIA is less frequently reported. The studies that categorized the stroke severity, suggested that disabling stroke had a higher incidence (58%) than non-disabling (26%) and transient ischemic attack (16%) (16). However, this data could be influenced by a lack of adequate and systematic neurological assessment to detect minor stroke or TIA in observational and randomized studies.

Initially, the results from PARTNER I trial (both cohorts A and B) showed greater stroke incidence in the group undergoing TAVI (1, 3, 17). Later, in the Corevalve trial with high-risk patient, patients undergoing TAVI had a numerically lower stroke rate at 30-day and 1-year compared to SAVR (2). In the recent randomized trials with intermediate risk patients, the results of the NOTION I, PARTNER-2, and SURTAVI trials showed a 1.4, 5.5, 3.4% 30-day stroke rate in the TAVI arm, compared to 3.0% (*p* = 0.37), 6.1% (*p* = 0.57), and 5.6% (95%CI −4.2 to 0.3) in the surgical arm, respectively. In addition, in the propensity matched comparison of the surgical arm from PARTNER 2 with the observational cohort of Sapien 3 study, the 30-day stroke rate was lower in the TAVI group (−3.5, 95% CI: −5.9 to −1.1, *p* = 0.004) (18). Thus, the initial fear of higher CVE rates in the TAVI arm has changed over time and now there is enough evidence to support that clinical CVE incidence is at least similar to the surgical arm (4, 8, 19).

Several meta-analyses, including mostly observational studies, have determined the incidence of stroke following TAVI (6, 7, 20). Eggebrecht et al. (with 10,037 patients from 53 studies) reported a 30-day stroke rate of 3.3 ± 1.8%, with the majority being major strokes (2.9 ± 1.8%). More recently, Muralidharan et al. (with 29,043 patients from 34 studies) and Auffret et al. (with 72,318 patients from 64 studies) reported a median 30-day stroke rate of 3.1 and 3.3%, respectively (9, 18).

## Temporal presentation and pathophysiology of cerebrovascular events

**Figure 2:**
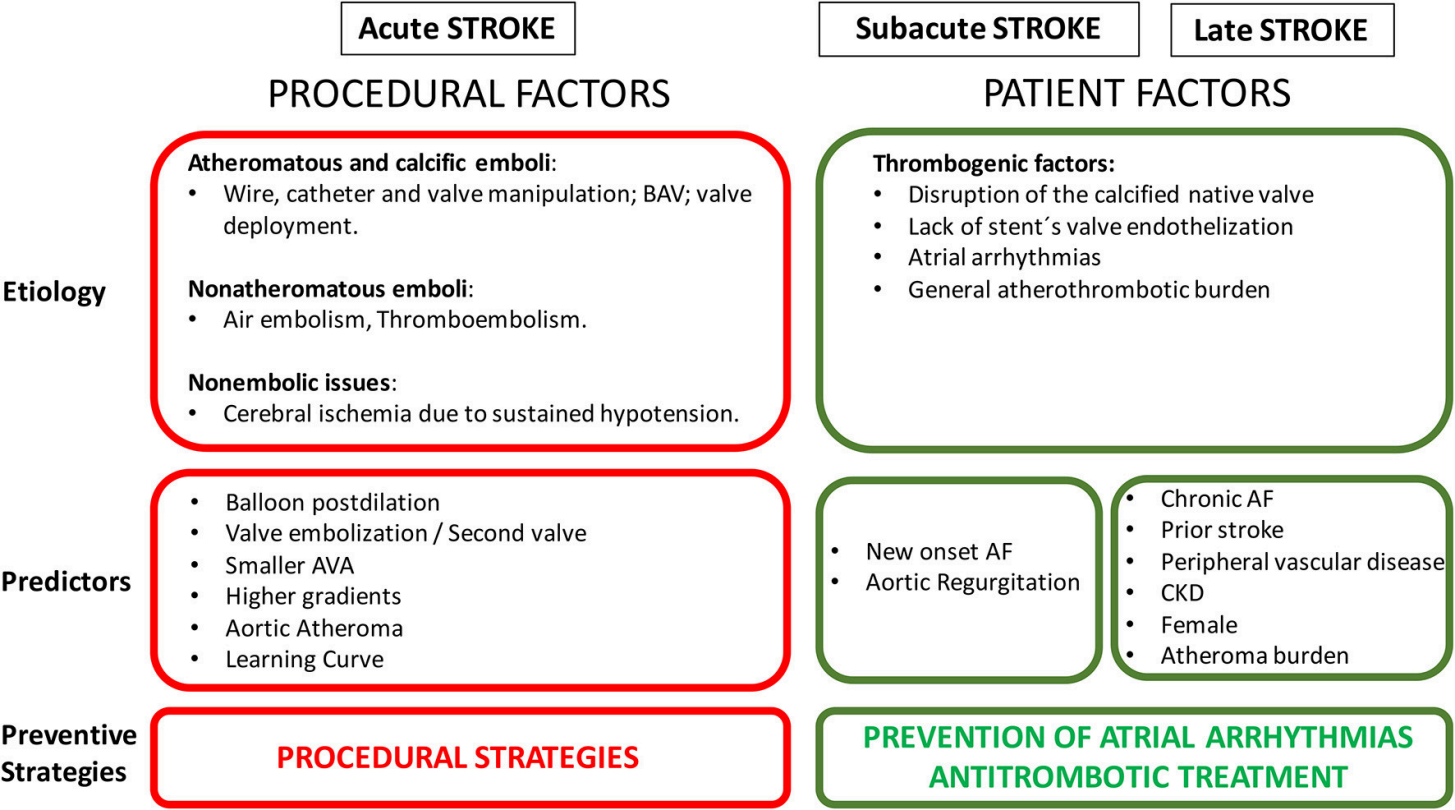
Risk factors of cerebrovascular events following TAVI. BAV, balloon aortic valvuloplasty; AF, atrial fibrillation; AVA, aortic valve area; CKD, chronic kidney disease.

Cerebrovascular events have been also classified according to the temporal pattern in acute (≤24 h), sub-acute (1–30 days), and late (>1 month) events. Several studies have shown that stroke incidence following TAVI has a peak in the immediate period after the procedure (24–48 h), reaching in some studies, half of the total events within 1 month (5, 21). Patients remain vulnerable for a period of up to 2 months after the procedure to subsequently decrease and stay stable over time. Temporal distribution of the CVE is closely related to their mechanism (Figure 2) (5).

### Acute cerebrovascular events

**Figure 3:**
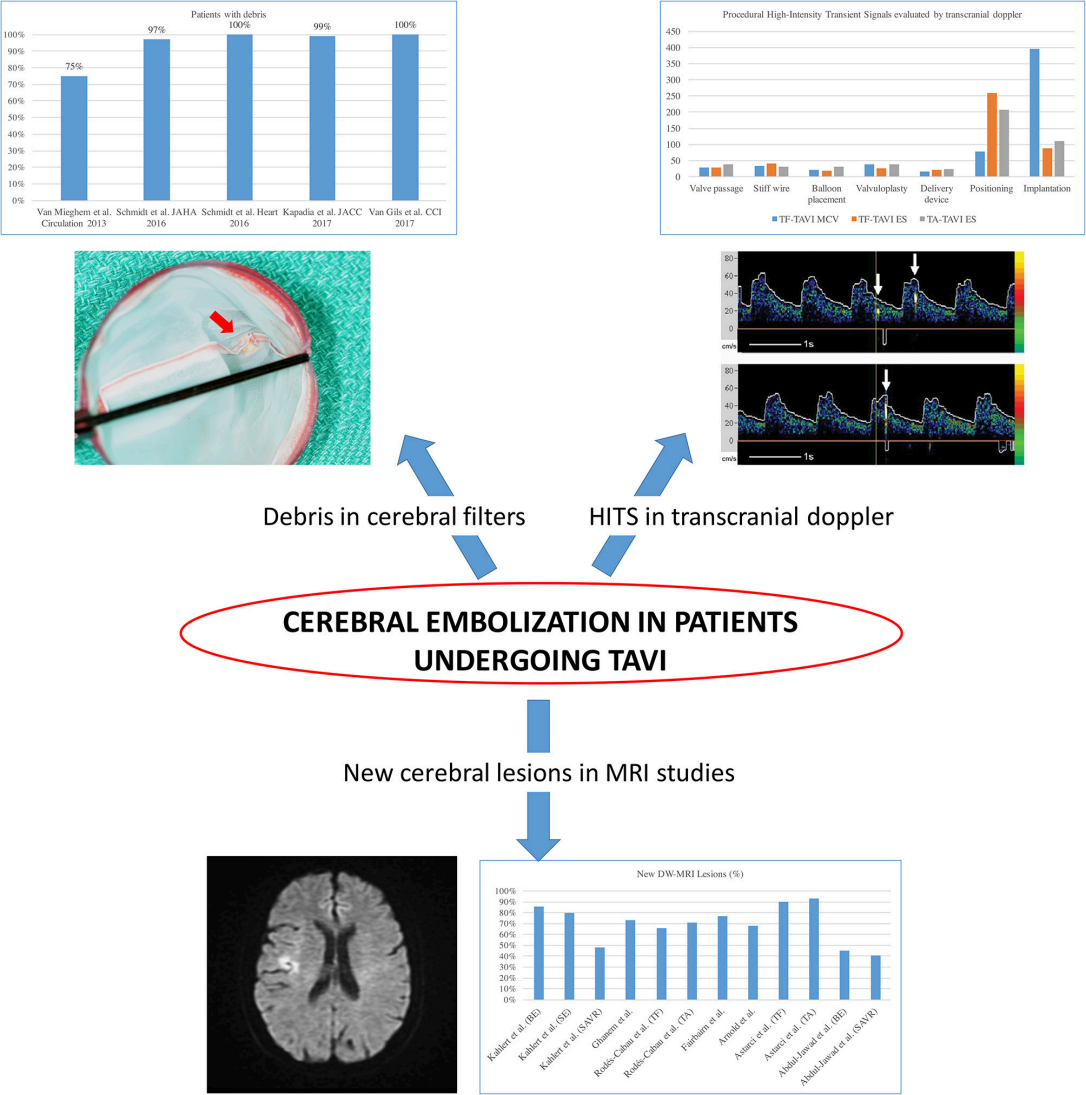
Evidence of cerebral embolization during and after transcatheter aortic valve implantation. HITS, high intensity transient signals; TAVI, transcatheter aortic valve implantation; DW-MRI, diffusion-weighted magnetic resonance imaging; TF, transfemoral; TA, transapical. Adapted from Abdul-Jawad Altisent et al. (23) and Kahlert et al. (24).

**Figure 4:**
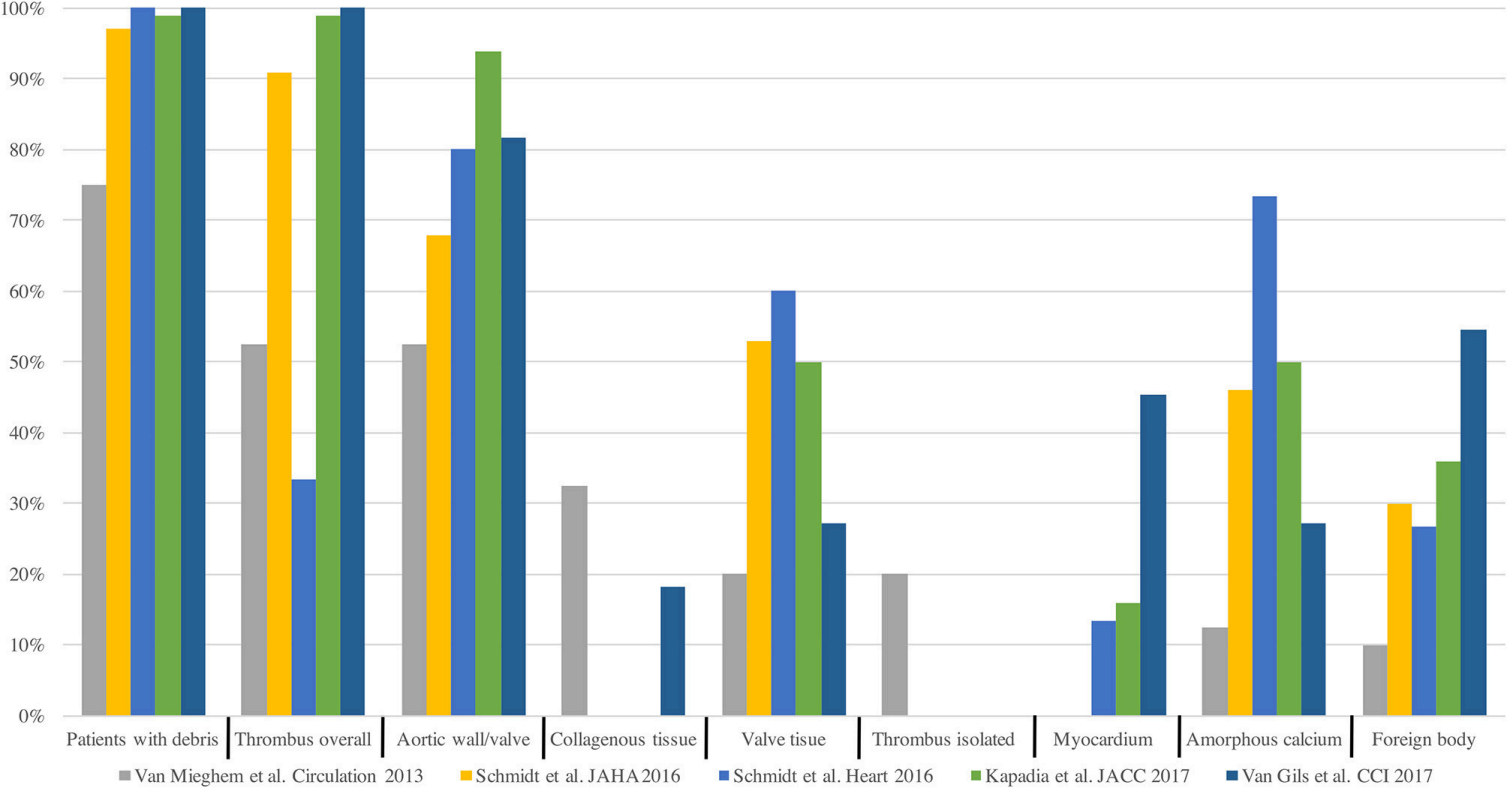
Frequency and distribution of captured debris in histopathologic analysis.

Most Acute CVE after TAVI are related to an ischemic origin, with <5% reported as hemorrhagic stroke (16). Most of this ischemic CVE are related to an embolic source. Due to the constitution of the calcified aortic valve and the walls of the aorta, its manipulation with rigid and large delivery catheters, balloon valvuloplasty or the interaction of the stent valve during the positioning or valve release will inevitably generate embolic material (22). This fact is supported by several different findings (Figure 3): Firstly, studies with diffusion-weighted magnetic resonance imaging (DW-MRI) demonstrated that between 60 and 90% of patients had new silent cerebral lesions after TAVI, independently of the vascular access or device type (22, 25, 26). These lesions were generally multiple, diffuse, distributed in both cerebral hemispheres and from both cerebral vascular territories (anterior and posterior) in most patients, suggesting an embolic nature. Secondly, procedural transcranial doppler studies confirmed that there were high intensity signals (HITS) in the middle cerebral artery in almost all the phases of the procedures, but especially during valve positioning and implantation (24). Interestingly, it has been suggested that valve design and implantation process could be associated with different temporal pattern of the HITS. While balloon expandable valve produces more emboli during valve positioning, self-expandable valve has greater amount of HITS during valve deployment (24). Thirdly, Van Mieghen et al., extensively examined the incidence and the histopathology of embolic debris retained in an embolic protection device during TAVI (27) (Figure 3). In the majority of cases (>85%), debris was obtained after the procedure, with a median size of 1 mm (IQR 0.6–1.6 mm). The nature of these emboli was varied. The most frequent was fibrin and thrombotic material (74% of the patients) (Figure 4) that were found in similar proportion in balloon and self-expanding valve. The wires and catheters used are known to be prothrombotic, associated with suboptimal anticoagulation during the procedure could be a potential source of thrombus formation. Additionally, damage to endothelium secondary to catheters manipulation, may cause platelet activation and the coagulation cascade, resulting in thrombus generation. Tissue-derived debris was present in 63% of the patients, with higher proportion in patients with balloon-expandable valve and higher degree of oversizing. This proportion of debris and its histopathology nature has been confirmed in more recent studies (27–31) (Figure 4). In this line, another study reported that total atheroma volume in the aorta was associated with higher risk of acute CVE (32). Another possible source of CVE during the procedure was air embolisms, especially associated with large delivery catheters and contrast injection. However, air embolisms are usually considered temporary and are difficult to detect.

Systemic hypotension could also develop cerebral hypoperfusion, especially in the border territories supplied by different cerebral arteries, causing a watershed infarct. At least, one ventricular rapid pacing is mandatory in almost every TAVI, either with the valvuloplasty or balloon postdilation (more frequently performed with self-expandable valves) or during valve implantation (with balloon-expandable valves). Rapid pacing causes an impairment of cerebral perfusion but it is usually transitory and well tolerated with a prompt recovery. Patients with very low ejection fraction, especially after a long ventricular rapid pacing, may have a prolonged period of hypotension that requires inotropic support. Additionally, permanent cerebral injury could be caused by maintained systemic hypotension in the setting of hemodynamic instability during any procedural complication (bleeding, cardiac tamponade, severe acute aortic regurgitation…), even when inotropic and mechanical circulatory support are provided. Fortunately, the incidence of such complications has clearly decreased in the later years.

### Subacute/late cerebrovascular events

Cerebrovascular events occurring more than 48 h after TAVI are unlikely to be related to the procedure *per se*. The etiology of delayed CVE is less understood and has a multifactorial origin. In the immediate period after valve implantation, several theoretical phenomena may be thrombogenic. Disruption of the calcified native valve with denudation of the endothelium, the stent of the valve before endothelization, and the paravalvular space with the native valve compressed against the aortic wall, are some examples of potential sources of thrombus. Intraartrial thrombus formation related to atrial arrhythmias could be another source of thromboembolism. Intracardiac thrombus, usually detected in left atrial appendage, and spontaneous echo contrast were frequent findings in patients with aortic stenosis (10 and 24%, respectively) (33).

## Predictors of cerebrovascular events after TAVI

Based on their mechanism, predictors of CVE following TAVI can be divided in procedural and patient factors related (Figure 2). Several studies reported predictors of developing neurological events after TAVI (5, 7, 34). Initially, the PARTNER trial showed that patients with lower aortic valve area had a higher risk of CVE in the early period after TAVI (16). This was related to a more calcified valve, with a plausible mechanism of higher risk of embolization. In this line, Nombela-Franco et al. reported that patients with a higher degree of valve calcification underwent more frequently balloon post-dilation (BPD) to treat paravalvular regurgitation (35). These patients also had a higher rate of acute CVE. However, it was not possible to determine if the independent factor for the acute CVE was the amount of calcium or BPD. Later, other studies highlighted the impact on acute CVE of mechanical procedural factors, such as number of implantation attempts, valve embolization, second valve implantation, or BPD (5, 14, 21).

Attempts have been made to find other risk factors related to CVE following TAVI, such as the presence of porcelain aorta, which in cardiac surgery is a well-established factor with a higher risk of stroke (36). Although porcelain aorta is associated with a greater burden of cardiovascular risk factors (37), and therefore could lead to a higher incidence of late CVE, the currently available evidence have not found a higher incidence of stroke in this group of patients undergoing TAVI compared to patients without porcelain aorta (1.6 vs. 2.5% respectively, *p* = 1.0) (37–39). It would appear reasonable that operator, and center experience may also be predictors of stroke post TAVI. Carroll et al. evaluated the association of hospital TAVI volume and patient outcomes by using data from 42,988 procedures conducted at 395 hospitals from the TVT Registry from 2011 through 2015. High-volume centers had significantly lower in-hospital events, but no difference was found in the stroke rate (*p* = 0.14) (40). However, a greater center experience was associated with lower stroke rates (2.03 vs. 1.66%, *p* = 0.01), similar findings described by Auffret et al. showing 1.55 fold more risk of CVE after TAVI during the first half of enrollment (95%CI, 1.16–2.08, *p* = 0.003) (7).

Regarding the access site, no differences were found in MRI studies comparing transfemoral vs. transapical approaches (41). Also, some meta-analysis have revealed that a non-transfemoral approach did not carry a higher stroke risk (RR 1.03, 95%CI 0.83–1.27, *p* = 0.81) (7). Transcarotid approach in terms of stroke risk is more controversial. Non-randomized trials found similar neurological outcomes (3.8% 30-day CVE) compared to an historically transfemoral cohort (42). However, recent evidence with a small number of patients (*n* = 22) showed more than twice the number and total volume of new ischemic lesions evaluated by diffusion-weighted magnetic resonance imaging within the left hemisphere (*p* < 0.01 for both) when performing TAVI through the left carotid artery (43). Therefore, more information is needed to clarify this issue.

In the sub-acute phase after TAVI, the strongest predictor for 30-day CVE found in several studies was NOAF, which usually occurs in an average of 15% of the patients. In a recent meta-analysis NOAF had a 1.85-fold increased 30-day hazard for CVE after TAVI. Although the incidence and definition of NOAF has varied across the studies, some studies reported that even short and transient periods of NOAF may have a significant influence in CVE, especially because some of these patients had a suboptimal anticoagulation regimen (44, 45). The stroke rate of patients with optimal anticoagulation was 2.9% compared to 40% in non-anticoagulated patients (45). Also Nuis et al. found a temporal relation between NOAF and CVE, where NOAF preceded the first signs of neurological impairment in all patients with an ischemic stroke (44). Auffret et al., also found that chronic kidney disease (CKD) defined by an estimated glomerular filtration rate <60 ml/min^.^1.73 m^2^, was an additional factor associated with an increased risk of 30-day CVE (7). Renal disease facilitates chronic inflammation, oxidative stress and atherosclerotic process with an increase in vascular calcification and endothelial dysfunction (46). In general, patients with renal impairment usually have an excess risk of stroke after adjusting for age and other cardiovascular risk factor (47).

## Prognosis value on mortality, morbidity and neurocognitive function of cerebrovascular events after TAVI

**Figure 5:**
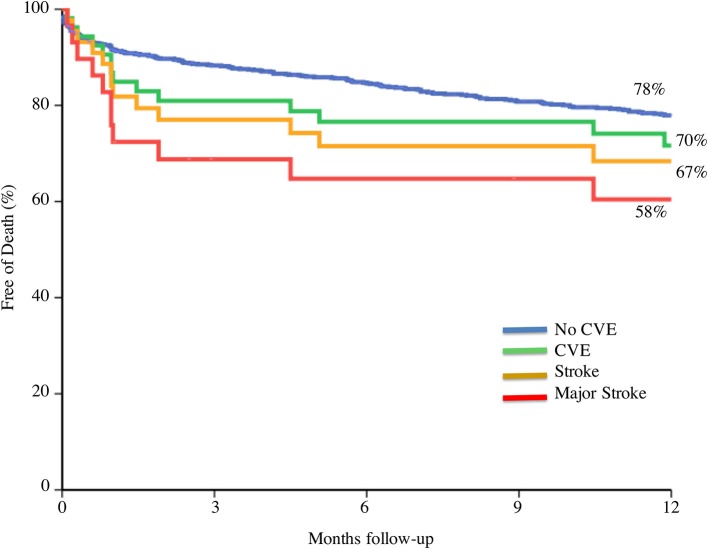
Prognosis value on mortality according to cerebrovascular event severity. CVE, cerebrovascular events. Adapted from Nombela-Franco et al. (5).

Patients with an early CVE (within 30-day) after TAVI had significantly higher mortality at 30-day and 1-year as compared to those without CVE, as shown in several studies (5, 9, 20, 21, 48–50). Eggebrecht et al. also reported a 3.5 times higher 30-day mortality after stroke in a large meta-analysis. One-month mortality was as high as 25% in patients with CVE compared to 7% in patients without CVE. Similarly, in a more recent meta-analysis of 29,034 patients, mortality was six-fold higher in patients with stroke (20). Short and long term mortality risk is incremental according to the severity of the CVE, being significantly higher with major stroke (OR 7.43; 95% CI, 2.45–22.53; *p* = 0.001, and HR 1.75; 95% CI, 1.01–3.04; *p* = 0.043 respectively) (Figure 5).

In addition, stroke is probably the most feared complication (even more than death) reported by patients (51). Coylewright et al. described that the majority of patients undergoing TAVI wanted to maintain independence and be able to participate in daily hobbies, and only 7% of the patients stated that their main goal was to stay alive after the procedure. Importantly, the total proportion of patients with a permanent disability (modified Rankin scale of 2–5) at 30-day is around 50% of patients with CVE (5, 48). This highlights the impact of major stroke, not only in mortality, but also in patients' quality of life.

### Cognitive function and cerebral lesions

As previously commented, new cerebral silent lesions are found in a high (~75%) percentage of patients undergoing TAVI, and cerebral embolization is almost ubiquitous in studies with filters embolic protection devices. The Rotterdam Scan Study evaluated the presence of silent cerebral infarction in a group of healthy elderly patients, demonstrating 3 times higher risk of stroke, greater decline in cognitive functions and 2 times more risk of dementia after a follow-up of almost 4 years (52, 53). In addition, in SAVR patients, it has been found an association between new cerebral lesions in DWI-MRI studies and cognitive deterioration during follow-up (54). However, the impact of these silent cerebral emboli and its relationship with cognitive deterioration after TAVI is under debate. In one study (*n* = 111), neurocognitive function declined in 5.4% of patients after TAVI (55) but new cerebral lesions were not associated with cognitive impairment. In a study of 44 consecutive patients with systematic baseline and serial neurologic and cognitive assessments combined with post-procedure DWI-MRI imaging, brain lesions were detected in 94% of the patients (56). Neurologic impairment, assessed by a worsening in the National Institutes of Health Stroke Scale (NIHSS), was detected in 21 and 11% of patients, at discharge and 30-day, respectively, and it was slightly higher in patients with cerebral lesions (23 vs. 15%). In addition, cognitive decline evaluated by the Montreal Cognitive Assessment was identified in 33 and 41% of patients at discharge and 30-day, respectively. However, many studies, failed to find an association between new cerebral lesions post-TAVI and cognitive impairment (57, 58). In more recent studies the volume of these new cerebral lesions had a weak, although statistical significant, correlation with neurocognitive changes (31). These discrepancies across studies could be explained due to the lack of validated models to assess neurocognitive function in TAVI candidates, a certain degree of cognitive dysfunction pre-procedural in some patients and the high prevalence of inter and intra-observer variability for these tests.

Several studies have analyzed the global impact of cognitive function after TAVI, independently of the presence of cerebral lesions. Schoenenberger et al. (*n* = 229) showed in a prospective analysis that cognitive function, assessed by the Mini-mental State Examination, worsened in 12.7% (*n* = 29) of patients. Interestingly among the patients with cognitive impairment before the procedure, TAVI was related to an improvement in the cognitive function in 37.5% (*n* = 18). Baseline smaller aortic valve areas were lower in patients who cognitively improved, suggesting a greater hemodynamic benefit in those patients (59). Another study evaluated changes of the Montreal Cognitive Assessment score with an improvement at the early stage and remained stable at 1-year (60). This global improvement was more pronounced among the 40% of patients with baseline cognitive impairment. However, early decline in some complex cognitive functions was also observed in 26%, persisting at 1 year in 10% of the patients. Thus, long-term follow up studies are needed to clarify the consequences of this nearly universal cerebral embolism imaging finding post TAVI in regards to neurocognitive impairment and vascular dementia, especially in younger patients with longer life expectancy.

## Prevention strategies

As previously commented, the majority of CVE following TAVI have an embolic origin. The strategy to obtain, at least a theoretical reduction of the CVE rate, is: (1) to decrease thrombus formation and debris embolization, and (2) once they have been formed or embolized, to avoid them reaching cerebral vasculature by using mechanical barriers such as embolic protection device (EPD). Regarding the first objective improving device performance and procedural technique (less damage of the aortic wall, less traumatic valve crossing and avoiding multiple recaptures and balloon pre and post-dilation) could lead to a significant reduction of the amount of debris. Another important factor is the antithrombotic therapy before, during and after the procedure.

## Antithrombotic therapy

Antithrombotic treatment in patients undergoing TAVI is currently one of the most important research scenarios in the TAVI field, with several large multicenter randomized clinical trials already ongoing. However, in the initial phase of the TAVI, and until definitive trials results, antithrombotic treatment has been recommended on an empirical basis. Guidelines do not recommend any treatment before the procedure (61, 62) and pre-procedural aspirin plus a loading dose of 300–600 mg of clopidogrel has been adopted from randomized clinical trials in patients undergoing transfemoral TAVI (1, 2, 19). Pre-procedural loading dose of clopidogrel is avoided in non-transfemoral cases. Most of the centers achieved intraprocedural anticoagulation with full-dose of intravenous heparin, although one fourth of the centers do not performed activated clotting time (ACT) measurement to guide anticoagulation (63). One non-randomized retrospective study compared the efficacy and safety of the standard bolus of heparin based on body weight vs. an adjusted dose of heparin guided by a baseline ACT. Interestingly, the ACT-guided group received lower total dose of heparin with no differences in terms of stroke and lower rate of major and life-threatening bleeding (64). The BRAVO trial reported that bivalirudin did not reduce the rates of major bleeding within 48 h or net adverse cardiovascular events at 30 days (65). In the MRI-substudy of the BRAVO trial, new post-procedural cerebral lesions and large lesions (volume ≥1,000 mm^3^) were also similar in both groups (66). Thus, bivalirudin was considered an alternative procedural anticoagulant in patients unable to receive heparin.

**Figure 6:**
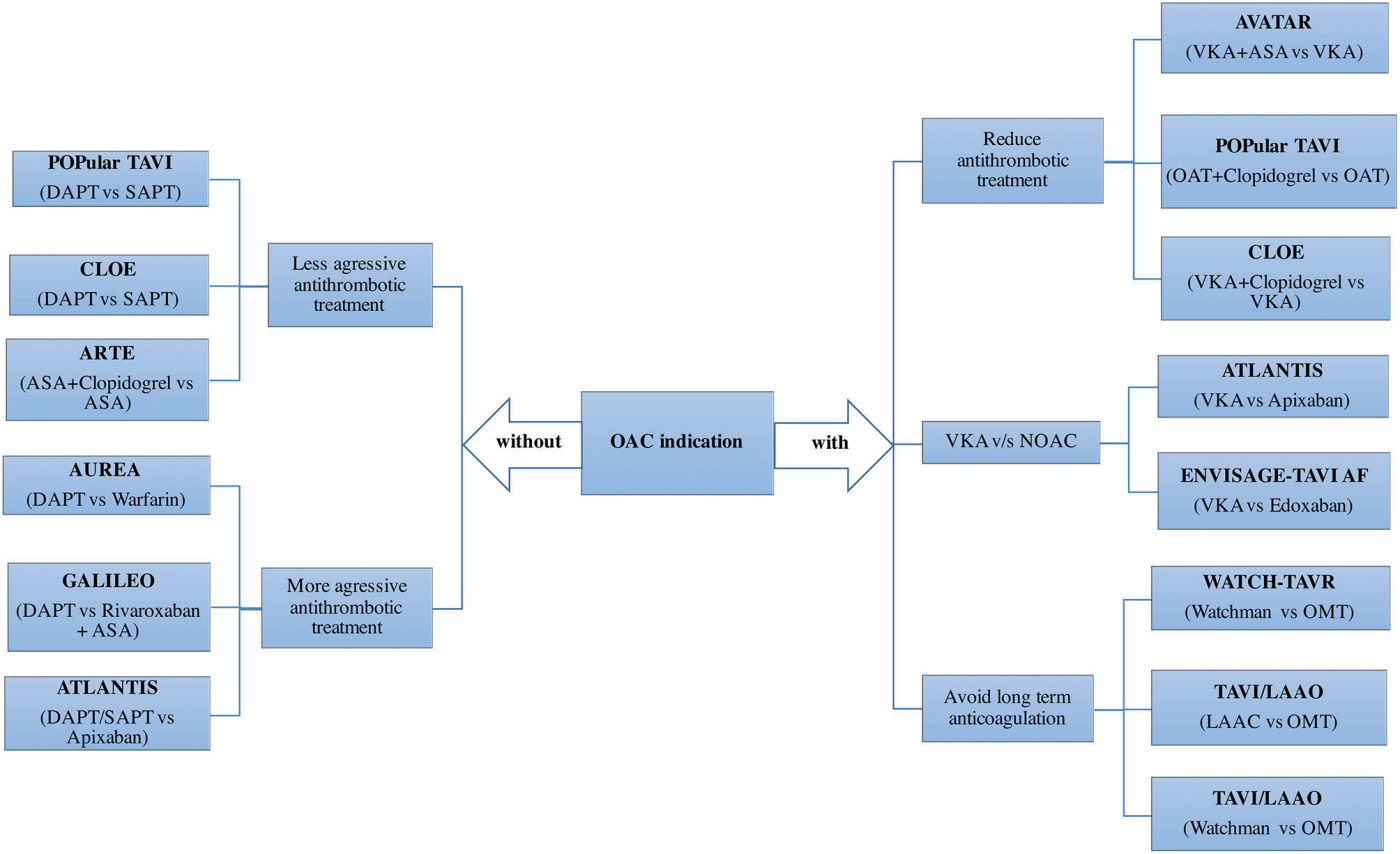
Adjunctive antithrombotic treatment after TAVI: Randomized clinical trials. ASA, acetylsalicylic acid; DAPT, dual antiplatelet therapy; LAAO, left atrial appendage occlusion; NOAC, novel oral anticoagulant; OAC, oral anticoagulant; OMT, optimal medical treatment; SAPT, single antiplatelet therapy; TAVI, transcatheter aortic valve implantation; VKA, vitamin K antagonists.

Regarding the antiplatelet treatment after the procedure, dual antiplatelet therapy (DAPT) is the most common antithrombotic treatment prescribed at hospital discharge in patients without AF, but with a high variability of the duration across centers (63), ranging from 1 to 12 months in most centers. Although a minority of centers initially adopted a single antiplatelet treatment, there are 3 small randomized trials suggesting no benefit of DAPT in terms of ischemic events with a higher rate of bleeding complications (67, 68). The CLOE and Popular TAVI trials will determine the efficacy and safety of a less aggressive antiplatelet treatment in patients undergoing TAVI (Figure 6). On the other hand, valve thrombosis and its relation to CVE (69), has raised the question whether a more aggressive antithrombotic treatment should be the preferred option in the first months after the procedure. Several on-going trials would help to clarify this issue (Figure 6).

In patients with AF, the variability in the antithrombotic treatment across centers is even greater. In an international survey with 250 centers, warfarin alone or combined with either ASA or clopidogrel were used in 28, 39, and 26% of the centers, respectively (63). Triple therapy (warfarin+DAPT, 4.5%) or left atrial appendage closure (0.5%) was marginally used as the standard care in patients with AF undergoing TAVI. Two observational studies showed no differences in terms of stroke, but lower bleeding rates in patients treated with warfarin alone compared to a combination of warfarin with one antiplatelet drug, especially with ASA (70, 71). Another interesting alternative in patients with AF is to mechanically close the left atrial appendage in order to reduce bleeding events without jeopardizing stroke protection (72). The impact of non-vitamin K antagonist oral anticoagulants and left atrial appendage closure would be tested in future randomized trials (Figure 6).

## Embolic protection devices

**Figure 7:**
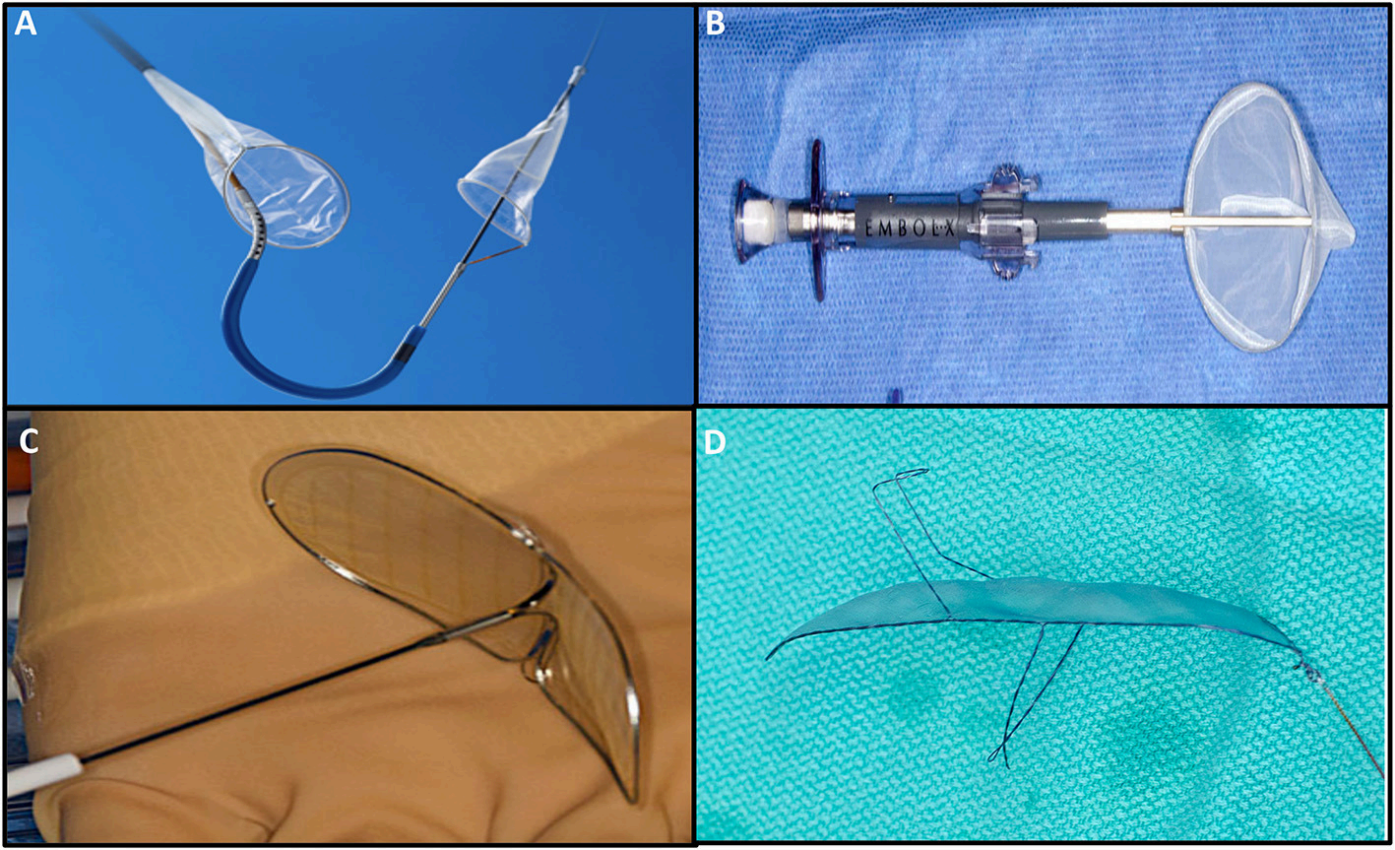
Cerebral embolic protection devices: **(A)** Sentinel (Claret Medical Inc., Santa Rosa, CA; **(B)** Embol-X (Edwards Lifesciences, Irvine, CA); **(C)** Embrella (Edwards Lifesciences, Irvine, CA); **(D)** TriGuard (Keystone Heart Ltd, Caesarea, Israel).

Embolic protection devices have emerged as a potential solution to decrease cerebral embolization and the associated neurological effects. To date, 4 types of EPD have been studied, with differences mainly in terms of design and access routes (Figure 7, Table 3). Deflectors, represented by the Embrella (Edwards Lifesciences, Irvine, CA) and TriGuard (Keystone Heart Ltd, Caesarea, Israel) devices are released along the external curvature of the aortic arch providing coverage to the innominate artery, common left carotid and in the case of the Triguard also to the left subclavian artery rejecting the embolized material toward the descending aorta. On the other hand, there are filter-type systems represented by Sentinel (Claret Medical Inc., Santa Rosa, CA) and Embol-X (Edwards Lifesciences, Irvine, CA). The first contains filters that are released in the brachiocephalic trunk and the left common carotid, and the second is positioned in the ascending aorta being deployed before aortic puncture for transaortic TAVI, providing a full cerebral coverage (73).

**Table 3:** Main characteristics of the embolic protection devices.

**Device**	**Manufacturer**	**Design**	**access**	**Delivery**	**deployment**
Embrella	Edwards Lifesciences, Irvine, CA	Deflector	Radial/Brachial	6F	Aortic arch
TriGuard	Keystone Heart Ltd, Caesarea, Israel	Deflector	Femoral	9F	Aortic arch
Sentinel	Claret Medical Inc., Santa Rosa, CA	Filter	Radial/Brachial	6F	1 filter to brachiocephalic trunk and 1 filter to left common carotid
Embol-X	Edwards Lifesciences, Irvine, CA	Filter	Direct aortic	14F	Ascending aorta

*Adapted from Steinvil et al. (73)*.

### Randomized clinical trials

The current available evidence in relation to EPD are constituted by a series of observational and 5 randomized studies (31, 58, 74–76), which have been also combined in several meta-analysis (77–80). Main limitations of randomized trials have been the relatively low number of patients included and using surrogate events such as the number and volume of cerebral lesions as the primary endpoint, instead of clinical neurological events.

The Embrella device was evaluated in the prospective non-randomized PROTAVI-C trial (*n* = 52) by DW-MRI (at baseline, 7 and 30 days) and procedural transcranial Doppler. Its implantation was associated with higher total number of HITS than the control group (*p* < 0.001). Both groups presented new brain lesions (100% of patients in each group), however the intervention group showed a lower volume of ischemic lesions compared to the control group (*p* = 0.003) (81).

The DEFLECT III multicenter randomized trial (*n* = 85) evaluated the TriGuard system, with neurocognitive assessment and DW-MRI at baseline, pre-discharge and 30-day. The safety endpoint (death, stroke, major bleeding, acute kidney injury stage 2 or 3, major vascular complication) occurred in 21.7% of the intervention group and in 30.8% of the control group (*p* = 0.34). Patients with a full cerebral coverage (89% in the intervention group), had a greater freedom from new ischemic brain lesions at 30-day (26.9 vs. 11.5%) and lower neurological deficit in NIHSS scale (3.1 vs. 15.4%; *p* = 0.16) (74).

There is very limited evidence in trans-aortic TAVI with the EMBOL-X device in a single randomized trial that included 30 patients (14 patients with filter). In the intervention group, a nonsignificant decrease in new brain lesions (57 vs. 69%; *p* = 0.70) and volume lesions (88 ± 60 mm^3^ vs. 168 ± 217 mm^3^; *p* = 0.27) in DW-MRI at 7 days post-procedure was found (75).

The MISTRAL-C multicenter randomized trial (*n* = 65) compared the number of new brain lesions evaluated by DW-MRI and neurocognitive function before and after TAVI (average 5 days) using the Claret Sentinel device. The primary endpoint (percentage of patients with new brain lesions) was not reduced in the device group (73 vs. 87%; *p* = 0.31) with a tendency to lower volume of new brain lesions (95 vs. 197 mm^3^; *p* = 0.171). A significant reduction in the number of patients with multiple brain lesions (20 vs. 0%; *p* = 0.03) and lower cognitive impairment (4 vs. 27%; *p* = 0.017) was observed. Regarding study limitations, images and neurocognitive tests were obtained in only 57 and 80% of patients with and without EP, respectively (76).

The CLEAN-TAVI randomized trial (*n* = 100) with the Claret Sentinel device, was the first trial to show a positive result in the primary end-point (new brain lesions evaluated by DW-MRI at 2 days after the intervention). The filter group was associated with a significant reduction of new cerebral lesions in the protected territories (4 vs. 10, *p* < 0.001) and in the entire brain (8 vs. 16, *p* = 0.002). Volume of these lesions was also lower in the filter group (466 vs. 800 mm^3^; *p* = 0.02), with a total of 5 minor strokes in each treatment arm (54–58, 68–70).

The SENTINEL study (*n* = 363) is the largest randomized study with EPD. The device was successfully implanted in all the patients, and obtained almost universally embolic material, mostly non-thrombotic from the arterial walls. Fluoroscopic time was longer in the device group with a non-inferior rate of the primary safety end-point (7.3% vs. 9.9, *p* = 0.41). Primary efficacy end-point (volume of new cerebral lesions) was similar in both groups (102.8 vs. 178 mm^3^; *p* = 0.33) in the DW-MRI performed between 2 and 7 days after the procedure. The stroke rate was numerically lower in the device group (5.6 vs. 9.1%, *p* = 0.25) (31).

Several meta-analyses have combined the results of the observational and randomized studies. Surrogate end-point such as the number and volume of new brain lesions seemed to be reduced in favor of the EPD (77, 80), although differences in the global rate of stroke or death is more controversial. While, some meta-analyses did not show any an statistical differences, other showed a reduction in the combined event of death or stroke using EPD, performing an analysis by the fixed-effects method (79). Finally, a recent single-center observational study, included 280 consecutive patients treated with the Sentinel device and compared them to a historical cohort of patients (*n* = 522) treated in an identical setting but without a filter. After a propensity score matching (*n* = 280 in each group), patients in the filter group had a significant reduction of the stroke rate (1.4 vs. 4.6%, *p* = 0.03), or a combination of death or stroke (2.1 vs. 6.8%, *p* = 0.01) (82). The procedure without an EPD was the only independent predictor (*p* = 0.04) for the occurrence of stroke within 7 days.

### Cost effectiveness analysis

A stroke can have an unpredictable and devastating impact, not only in terms of mortality but also in terms of its sequelae (50% permanent disability). It is estimated that more than half of patients with a clinical stroke will be unable to return to work, and 1 in 3 patients will have serious financial problems (83–85). The economic and social impact of presenting a stroke after the implantation of TAVI is a topic to consider. It is estimated that during the index hospitalization, it can increase the costs of the initial hospitalization ~$25,000, with an average of 7 additional days of hospital stay compared to patients who do not have a stroke (86). This cost can be even higher in patients discharged with a moderate disability, in whom the annual health costs can be increased by up to $60,000 (87). According to meta-analyses from Giustino et al. 22 patients have to be treated to reduce one stroke or death using EPD (79). The Sentinel device has a cost around to $2,800, therefore, making a quick and simplified calculation, a total of $61,600 has to be spent to prevent one stroke or death, a value that may be justifiable given the negative physical, emotional and economic impact of stroke. However proper studies about the cost-effectiveness of EPD are needed to determine the validity of this rough calculation.

## Conclusions

Cerebrovascular events after TAVI had a multifactorial etiology with an incidence about ~3–4%. This complication has clearly a significant impact on patient's morbidity and mortality, mainly during its acute and subacute phase. Despite the fact that its incidence has slightly decreased in the modern TAVI era with greater knowledge and new technologies, it seems that cerebral embolization is ubiquitous after TAVI, proven by HITS during the procedure; new cerebral lesions on DW-MRI studies and debris captured in cerebral filters devices. The clinical impact of cerebral embolization is still under discussion. The currently available trials with EPD have not been designed to detect clinical CVE and they have assessed neurological damage by surrogate end-points such as rate or volume of new brain lesions. However, the expansion of the technique to younger and low risk patients will force us to look for new and better tools to avoid cerebral embolization.

## Author contributions

Each author has contributed to this work as follows: GA and LN-F: conception and design, drafting and revising of manuscript, final approval of the manuscript submitted; GT-C: critical review of the manuscript for important intellectual content; final approval of the manuscript submitted.

### Conflict of interest statement

LN-F has served as a proctor for Abbott; and has received speaker honoraria from Edwards Lifesciences Inc. The remaining authors declare that the research was conducted in the absence of any commercial or financial relationships that could be construed as a potential conflict of interest.
